# New Insights into the Interaction of Carbohydrate and Fat Metabolism During Exercise

**DOI:** 10.1007/s40279-014-0154-1

**Published:** 2014-05-03

**Authors:** Lawrence L. Spriet

**Affiliations:** Department of Human Health and Nutritional Sciences, University of Guelph, Room 354, ANNU Building, Guelph, ON N1G 2W1 Canada

## Abstract

Fat and carbohydrate are important fuels for aerobic exercise and there can be reciprocal shifts in the proportions of carbohydrate and fat that are oxidized. The interaction between carbohydrate and fatty acid oxidation is dependent on the intracellular and extracellular metabolic environments. The availability of substrate, both from inside and outside of the muscle, and exercise intensity and duration will affect these environments. The ability of increasing fat provision to downregulate carbohydrate metabolism in the heart, diaphragm and peripheral skeletal muscle has been well studied. However, the regulation of fat metabolism in human skeletal muscle during exercise in the face of increasing carbohydrate availability and exercise intensity has not been well studied until recently. Research in the past 10 years has demonstrated that the regulation of fat metabolism is complex and involves many sites of control, including the transport of fat into the muscle cell, the binding and transport of fat in the cytoplasm, the regulation of intramuscular triacylglycerol synthesis and breakdown, and the transport of fat into the mitochondria. The discovery of proteins that assist in transporting fat across the plasma and mitochondrial membranes, the ability of these proteins to translocate to the membranes during exercise, and the new roles of adipose triglyceride lipase and hormone-sensitive lipase in regulating skeletal muscle lipolysis are examples of recent discoveries. This information has led to the proposal of mechanisms to explain the downregulation of fat metabolism that occurs in the face of increasing carbohydrate availability and when moving from moderate to intense aerobic exercise.

## Introduction

It has been known for many years that both carbohydrate and fat are important substrates for oxidative phosphorylation and energy production in skeletal muscle [1, 2]. The oxidation of any one fuel at rest or during exercise does not occur in isolation, and many aspects of metabolism are simultaneously active at a given point in time. Both carbohydrate and fat are oxidized at rest to provide the energy required for basal metabolic processes in skeletal muscle, and there is a reciprocal relationship between the utilization of carbohydrate and fat. Fuel shifts occur at rest despite a generally unchanged metabolic demand and are largely driven by the availability of substrate. For example, increasing the availability of blood glucose increases the uptake and oxidation of carbohydrate in skeletal muscle, while decreasing the availability and oxidation of fat, with little change in the metabolic rate.

During exercise, the metabolic rate and need for energy increases several fold over the resting rate, and the metabolic pathways that oxidize both fat and carbohydrate must be activated simultaneously [1, 2]. However, once a so-called ‘steady state’ at a given aerobic exercise intensity and metabolic demand has been established, there can be reciprocal shifts in the proportion of carbohydrate and fat that are oxidized [3]. The interaction between carbohydrate and fatty acid oxidation at a given exercise intensity is dependent on the intracellular and extracellular metabolic environments. The availability of substrate, both from inside and outside of the muscle, and the exercise intensity and duration will affect these environments.

This brief review examines how skeletal muscle regulates the proportion of fuel that is derived from carbohydrate and fat. The focus is on information obtained in human skeletal muscle, but findings from other mammalian skeletal muscle models are cited when human information is lacking. While descriptive studies documenting changes in the proportion of fat and carbohydrate utilization are numerous, the mechanisms regulating these fuel shifts have not been thoroughly elucidated. The review first discusses the classic studies that attempted independently to alter fuel availability and examine the alterations in the intracellular and extracellular environments that accounted for shifts in fuel use. It seems clear that these mechanisms are not mutually exclusive, and it is impossible to alter fuel availability independently without changing the intracellular metabolites that regulate key enzymes in the cell. Nevertheless, this approach yielded valuable insight into the interaction of carbohydrate and lipid metabolism at rest and during exercise. The review then examines recent work in which the focus has been on the emergence of detail relating to the complex regulation of fat metabolism in skeletal muscle. This research examines how increasing the availability of carbohydrate and increasing the exercise power output from approximately 40–60 % maximal oxygen uptake (*V* O_2max_) to approximately 70–85 % *V* O_2max_ decreases the reliance on fat oxidation.

## Classic Glucose–Fatty Acid Studies

The concept of a reciprocal relationship between fat and carbohydrate oxidation in muscle resulted from the work of Randle and colleagues in the 1960s [4–6]. The relationship was called the “glucose–fatty acid (G–FA) cycle.” Their early experiments examined the regulation of fuel use in artificially perfused and contracting heart muscle, and incubated, resting diaphragm muscle from rodents. These experiments demonstrated that increased lipid availability in the form of plasma free fatty acids (FFAs) increased fat oxidation and decreased carbohydrate oxidation in muscle, and that changes in the intracellular environment were responsible for the downregulation of carbohydrate use.

**Fig. 1:**
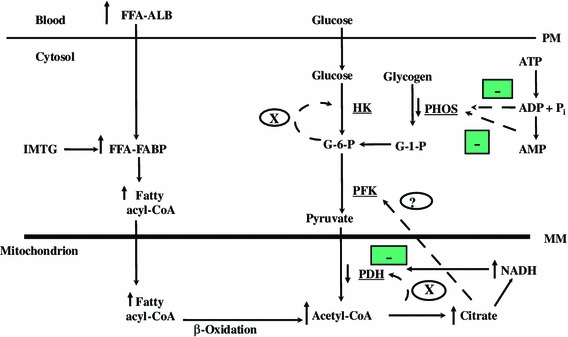
A contemporary view of the reciprocal relationship between carbohydrate and fat oxidation during exercise at power outputs of 40 %, 65 %, and approximately 80 % maximal oxygen uptake (*V* o_2max_). Increasing the availability of plasma free fatty acids (FFAs) had no effect on acetyl-coenzyme A (CoA) and glucose-6-phosphate (G-6-P) contents (X = no effect) at any power output and increased citrate content only at 40 and 65 % *V* o_2max_. Reduced FFA availability did reduce pyruvate dehydrogenase (PDH) activity at 40 and 65 % *V* o_2max_ and the flux through glycogen phosphorylase (PHOS) at all power outputs. The effect on phosphorylase flux was dominant at approximately 80 % *V* o_2max_ and was less important at 40 and 65 % *V* o_2max_. The accumulation of free adenine diphosphate (ADP), adenine monophosphate (AMP) and inorganic phosphate (P_i_) was reduced during exercise (as indicated by *dashes*) in the presence of increased FFA availability. Mitochondrial nicotinamide adenine dinucleotide (NADH) may be more abundant with high fat provision at the onset of exercise, increasing the aerobic production of adenosine triphosphate (ATP) and reducing the mismatch between ATP demand and supply and accounting for the reduced accumulation of ADP, AMP, and inorganic phosphate. *ALB* albumin, *FABP* fatty acid binding protein, *G-1-P* glucose-1-phosphate, *HK* hexokinase, *IMTG* intramuscular triacylglycerol, *MM* mitochondrial membrane, *PFK* phosphofructokinase, *PM* plasma membrane

The increase in fat availability resulted in key cellular changes including increased contents of muscle acetyl-coenzyme A (CoA), citrate, and glucose-6-phosphate (Fig. 1). These cellular changes downregulated carbohydrate metabolism at key regulatory sites. Test-tube (in vitro) work had previously established that acetyl-CoA inhibited the activity of the mitochondrial enzyme pyruvate dehydrogenase (PDH), by activating PDH kinase, the enzyme that phosphorylates PDH and moves it to its less active form. Therefore, a rise in acetyl-CoA decreased PDH activity and pyruvate oxidation and shifted fuel preference away from carbohydrate and towards fatty acids. Other in vitro work demonstrated that citrate was a potent inhibitor of the cytoplasmic enzyme phosphofructokinase, such that increases in citrate content would decrease carbohydrate use. Finally, glucose-6-phosphate had also been shown to inhibit hexokinase in vitro, making it difficult to move glucose into the muscle cell. By combining the findings from their isolated muscle experiments with the test-tube enzyme studies, Randle and colleagues [5, 6] established the mechanistic basis for their G–FA cycle, which explained how increased fat availability downregulated carbohydrate oxidation.

These experiments were performed by comparing the extremes of fat availability, with no fat in the perfusing medium compared with very high fat availability (0 vs. 1.8 mmol/L FFAs) while the availability of glucose was held constant. The philosophy was that carbohydrate was the default fuel of the muscles and when fat was available it was used and spared some carbohydrate. The goal was to explain the mechanisms responsible for carbohydrate downregulation and this was achieved. However, the so-called reverse experiments, in which fat availability was constant and glucose availability was altered, were not performed, as it was believed at the time that little regulation of fat metabolism in skeletal muscle existed. The key aspects in determining fat oxidation were simply thought to be the availability of fat and regulation at the level of the mitochondrial membranes through the carnitine–palmitoyl transferase (CPT) complex. While the mechanisms proposed in those early studies were sound, the question arose as to whether the findings were applicable to the skeletal muscles involved in whole body exercise in humans.

## Applicability to Human Skeletal Muscle?

The early experiments that led to the formation of the G–FA cycle concept were performed with contracting heart or resting diaphragm muscle from rodents, while perfused with, or bathed in, a medium that contained either no FFA or very high FFA concentrations [FFA]. In addition, the continuous duty-cycle of the heart and diaphragm muscles dictates that the majority of the fuel oxidized by these muscles has to be delivered to the muscles (carbohydrate from the liver and FFAs from adipose tissue). While there is glycogen and intramuscular triacylglycerol (IMTG) in these rhythmically contracting muscles, they do not provide a significant portion of the oxidized fuel [7]. In general, all skeletal muscles from rodents have lower IMTG and glycogen stores and rely more on fuel from outside the cell than in human skeletal muscle. In contrast, most human skeletal muscles rely more heavily on IMTG and carbohydrate from glycogen, and this is especially true during moderate- and high-intensity whole body aerobic exercise. Therefore, it was not known whether the mechanisms proposed by Randle [4] to explain the G–FA cycle in heart and diaphragm muscles would be transferable to peripheral human skeletal muscles during exercise.

## Altering Fat Availability

### Increasing Free Fatty Acid (FFA) Delivery

Subsequent human research in the past 50 years generally supported the notion of a reciprocal relationship between carbohydrate and fat oxidation in skeletal muscle, but not exclusively by means of the mechanisms defined in the original work by Randle and colleagues [5, 6]. Most human studies attempted to increase or decrease the plasma FFA availability, without affecting many other processes. While many models have been used with varying success, including high-fat meals and diets, short- and long-term aerobic training, caffeine administration, nicotinic acid ingestion, fasting, and prolonged dynamic exercise, the acute infusion of a lipid solution coupled with heparin administration has been most commonly and effectively used. This technique has the advantage of acutely (<30 min) increasing the plasma [FFA] without changes in the availability of other substrates or alterations in metabolite and hormone levels [8–10]. In contrast, dietary attempts at acutely increasing the availability of FFAs to the working muscles in humans immediately before or during exercise in an attempt to spare carbohydrate have been largely unsuccessful. This is due to the fact that fat is not digested quickly, and therefore prolonged alterations in the normal diet are required to alter the IMTG stores and spare carbohydrate. As a result, these practices are not generally in use by athletes.

If increasing fat availability decreases carbohydrate oxidation in human skeletal muscle, it would be expected that the inhibition of carbohydrate use would target the key sites regulating carbohydrate metabolism and oxidation (Fig. 1). These sites would include glucose transport (GLUT 1, 4) across the muscle membrane, glucose phosphorylation (hexokinase), glycogenolysis [glycogen phosphorylase (PHOS)], glycolysis (phosphofructokinase), and conversion of pyruvate to acetyl-CoA (PDH). These enzymes (at least PHOS, phosphofructokinase, and PDH) have been shown to be regulated by calcium, adenosine diphosphate (ADP), adenosine monophosphate (AMP), and inorganic phosphate both through direct (allosterically) and/or indirect (phosphorylation) regulation.

During exercise at approximately 80 % *V* O_2max_ in moderately active individuals, the majority of energy is derived from carbohydrate use and particularly from muscle glycogen during the first 20–30 min. Exercising at this high intensity in the presence of artificially elevated FFA levels decreased net glycogen use by approximately 50 % in the initial 15 min of exercise and increased fat oxidation by approximately 15 % during 30 min of exercise [8, 10]. The muscle contents of free ADP and AMP, activators of PHOS, were significantly reduced (increased less) in the high FFA condition during exercise, and appeared to explain the decreased PHOS activity and glycogen use. It was suggested that the mitochondrial reduced form of nicotinamide adenine dinucleotide was more abundant with high fat provision during the onset of exercise, increasing the aerobic production of adenosine triphosphate (ATP) and reducing the mismatch between ATP demand and supply, and accounting for the reduced accumulation of ADP, AMP, and inorganic phosphate [8, 9, 11]. There were no effects on muscle citrate, acetyl-CoA, and glucose-6-phosphate contents or the proportion of PDH in the active form (PDHa) [8, 9], and whole body glucose disappearance (glucose uptake) was also unaffected by elevated FFAs [10]. Therefore, at this intense aerobic power output in human skeletal muscle, the fat-induced downregulation of carbohydrate oxidation was controlled at the level of PHOS. The original work on the G–FA cycle by Randle and colleagues [4–6] did not include PHOS, because the diaphragm and cardiac muscles relied almost exclusively on exogenous substrates and instead focused on enzyme regulation downstream from PHOS, namely hexokinase, phosphofructokinase, and PDH.

When these experiments were repeated at lower exercise power outputs (~40 % and 65 % *V* O_2max_), as well as during dynamic knee extension, high fat provision again downregulated carbohydrate oxidation, suggesting this fuel reciprocity was not dependent on exercise intensity [11]. While the mechanism(s) of action responsible for the shift in fuel utilization again involved PHOS activity, small increases in citrate and lower PDHa levels suggested that downregulation was also present at phosphofructokinase and PDH at lower power outputs [11]. The increase in citrate supported one of Randle’s original hypotheses, but subsequent in vitro studies that examined the inhibitory effects of citrate on phosphofructokinase activity suggested that the small increase in citrate in the high-fat trials would have minimal inhibitory effects on phosphofructokinase in contracting human skeletal muscle, and probably is not a mechanism to shift fuel preference [12].

### Decreasing FFA Delivery

It is possible to take the opposite approach and decrease the availability of plasma FFAs while exercising at approximately 60 % *V* O_2max_ by ingesting nicotinic acid. In this situation, the respiratory exchange ratio, glycogen use (trend only), and PDHa were all higher than in the normal fat availability trial [13]. However, there was no effect on metabolic byproducts typically associated with the G–FA cycle, namely muscle citrate, acetyl-CoA, or pyruvate contents. In addition, there were no changes in free ADP and AMP to account for the higher glycogen breakdown and oxidation in the low-fat trial.

### Altering Intramuscular Triacylglycerol (IMTG) Content

Another question is whether increasing IMTG (a common aerobic training adaptation) decreases carbohydrate oxidation during exercise? The most common method to alter the IMTG availability is by long-term dietary manipulation. IMTG can be increased by 50–80 % following the consumption of high-fat diets in which fat supplies 50–70 % of the total energy intake and IMTG can be decreased when dietary fat intake is reduced from 22 to 2 % of energy intake [14, 15]. These long-term high-fat diets reduced muscle glycogen utilization and total carbohydrate oxidation rates during moderate intensity exercise, without altering glucose uptake [16]. Conversely, when IMTG is reduced following a low-fat diet (2 % of total energy intake), whole-body carbohydrate oxidation and muscle glycogen utilization are also increased without altering whole-body glucose uptake [14]. These data suggest that IMTG has no effect on muscle glucose uptake during exercise, but does influence muscle glycogen utilization. However, low-fat diets also contain high carbohydrate content, and therefore while IMTG contents are reduced, glycogen levels are increased. The opposite is also true as high-fat diets lead to low muscle glycogen stores. Therefore, to examine the pure interaction between the IMTG store and carbohydrate fuel metabolism, studies must use interventions that induce acute changes in IMTG content independent of alterations in the availability of glycogen (and other substrates such as plasma FFAs).

Interestingly, Burke et al. [17] demonstrated that the effects of a high-fat diet on reducing glycogen use during dynamic exercise persisted, even after muscle glycogen stores were returned to normal levels. In this paradigm, participants consumed either a high carbohydrate diet (9.6 g/kg/day carbohydrate and 0.7 g/kg/day fat) or an isoenergetic high-fat diet (2.4 g/kg/day carbohydrate and 4.0 g/kg/day fat) for 5 days while undergoing aerobic training. On the sixth day all participants consumed a high carbohydrate diet that normalized muscle glycogen levels, before an exercise trial on day 7. Regardless of the ‘normalized’ glycogen level, a portion of the ‘carbohydrate-sparing’ effect of the high-fat diet was still present during the day 7 exercise trial. The subjects in this study were well-trained athletes who continued to exercise at a very high level during the 5-day high-fat/low carbohydrate diet intervention. Typical high-fat dietary interventions are not associated with continued and uncompromised training, and therefore the ability to maintain and/or increase fat oxidation during aerobic training while consuming a high-fat diet may be extremely important for inducing these metabolic shifts. At the present time, the mechanisms responsible for the persistent effects of the high-fat diet are not known, but it is reasonable to speculate that a redistribution of fatty acid transport proteins to the plasma and mitochondrial membranes may contribute, but this has not been examined [15]. Interestingly, performance has also been assessed in these studies and the high-fat diets did not improve performance, even when a day of carbohydrate restoration occurred. Consequently, these dietary manipulations are not in use by elite athletes.

## Altering Carbohydrate Availability

While supplementing with fat in the days and hours before exercise and during exercise is not practiced by athletes, it is common practice with carbohydrate. However, in contrast to the numerous studies that have altered fat availability and examined the effects on the regulation of carbohydrate metabolism, there is little information regarding the influence of altering blood glucose and muscle glycogen availability on the regulation of fat metabolism. This has undoubtedly been due to our poor understanding of the regulation of skeletal muscle fat metabolism. Many physiological studies have pointed to potential regulatory sites of fat metabolism in skeletal muscle, but the understanding of these regulatory sites has only started to emerge.

## The Regulation of Fat Metabolism: A New Era

It has been known for some time that the regulation of adipose tissue lipolysis and the release of FFAs from adipose tissue and ultimate delivery to the muscle and the entry of fat into the mitochondria were important sites of control for fat oxidation. However, it is now understood that the regulation of fat metabolism and oxidation in skeletal muscle is as complex as carbohydrate metabolism and involves multiple regulatory sites, including (1) FFA transport across the muscle membrane with protein carriers; (2) binding and transport of FFAs in the cytoplasm; (3) IMTG synthesis; (4) IMTG degradation; (4) FFA transport across the mitochondrial membranes with the CPT complex and additional protein facilitators; (5) potential regulation within the β-oxidation pathway; and (6) the overarching aspect of the regulation of skeletal muscle fat oxidation—that the mitochondrial volume (the total amount of fat transport and metabolizing proteins) determines the overall capacity to oxidize fat [2, 3, 18–22]. There is also regulation in the tricarboxylic cycle and the electron transport chain that is common to both carbohydrate and fat.

### Increased Glucose Availability/Insulin Concentration Before and During Exercise

Several studies have demonstrated that increasing the muscle glycogen content before exercise, and the availability of exogenous carbohydrate before and during dynamic exercise, increases carbohydrate oxidation and reciprocally decreases fat oxidation [14, 23–25]. The carbohydrate-induced increase in plasma insulin concentration exerts a powerful inhibitory effect on adipose tissue triacylglycerol lipase (ATGL) and hormone sensitive lipase (HSL), reducing the breakdown of triacyglycerol and decreasing the circulating plasma FFA concentration. This translates into reduced delivery of FFAs to the muscles during exercise and a decrease in FFA uptake and oxidation. For example, when exercising in the fasted state, adipose tissue lipolysis exceeded skeletal muscle fat oxidation, whereas when carbohydrate was ingested before exercise, plasma insulin was elevated, adipose tissue lipolysis was reduced, and whole-body fat oxidation decreased [23]. Pre-exercise carbohydrate ingestion also increased glycolytic flux and carbohydrate oxidation and reduced estimated plasma and IMTG-derived FFA oxidation rates [23]. The magnitude of the decrease in fat oxidation equaled the reduction in lipolysis, suggesting a limitation of FFA availability for fat oxidation. So, while this regulation occurs in the adipose tissue, it has a large impact on fat metabolism in skeletal muscle.

**Fig. 2:**
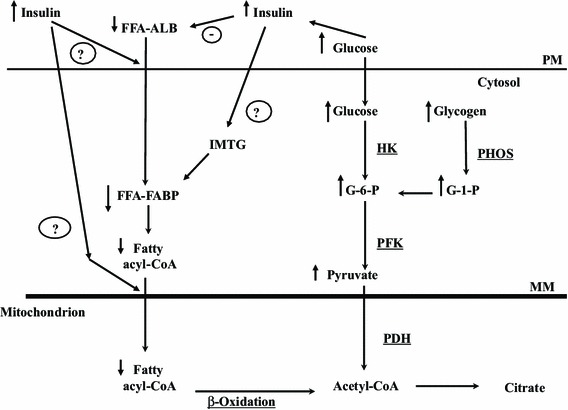
A schematic representation of the potential effects of carbohydrate ingestion before dynamic exercise in decreasing the plasma free fatty acid (FFA) concentration and downregulating fat metabolism in skeletal muscle. Ingested glucose increases the release of insulin, which inhibits adipose tissue lipolysis and reduces the plasma [FFA]. Increased insulin may also inhibit FFA transport across the plasma membrane (PM) and the mitochondrial membrane (MM) and decrease intramuscular triacylglcerol (IMTG) breakdown in skeletal muscle. Carbohydrate oxidation, from plasma glucose and/or muscle glycogen, is increased. *ALB* albumin, *CoA* coenzyme A, *FABP* fatty acid binding protein, *G-1-P* glucose-1-phosphate, *G-6-P* glucose-6-phosphate, *HK* hexokinase, *PDH* pyruvate dehydrogenase, *PFK* phosphofructokinase, *PHOS* glycogen phosphorylase

The same laboratory further examined the relationship between fat and carbohydrate oxidation by using two fatty acid tracers; the long chain fatty acid (LCFA) palmitate, which is dependent on muscle membrane and mitochondrial membrane transport for oxidation, and the medium chain fatty acid octanoate, which is not dependent on membrane transport [23]. Glucose ingestion during exercise reduced palmitate uptake and oxidation but did not reduce octanoate oxidation, suggesting that there was also an inhibitory effect of carbohydrate ingestion on FFA transport across the muscle and/or the mitochondrial membranes, resulting in reduced fat oxidation [23]. Therefore, the reduction in fat metabolism following glucose ingestion appears to be due to the combined effects of decreased adipose tissue FFA release and provision to muscle and to direct inhibitory effects on fatty acid oxidation in the muscle. One possibility to explain these results could be that increased insulin directly inhibits the transfer of fat across the muscle membrane and/or the mitochondrial membranes and also inhibits IMTG lipolysis, while at the same time stimulating the esterification of incoming FFAs (Fig. 2). While there has been little done on the regulation of IMTG hydrolysis in human skeletal muscle, HSL activity was reduced when carbohydrate was ingested during exercise [26]. There is no information regarding whether the newly identified ATGL [27] is affected by insulin, but ATGL content increased twofold in human skeletal muscle following 8 weeks of exercise training [28]. There may also be regulation at these sites by glycogen or glycolytic intermediates, but there is currently little evidence to support this. While an elevated insulin concentration may exert these effects following glucose ingestion before exercise (Fig. 2), it does not explain the increase in carbohydrate oxidation and decrease in fat oxidation that occurs when the power output increases from approximately 40–60 % *V* O_2max_ to approximately 70–85 % *V* O_2max_ during exercise.

### Fat Oxidation is Downregulated at Higher Aerobic Exercise Intensities

Fat oxidation increases from rest to low- and moderate-intensity exercise (maximum at ~60–65 % *V* O_2max_), but decreases at power outputs above approximately 75 % *V* O_2max_ [29–31]. Increasing the exercise power output above approximately 50 % *V* O_2max_ also increases the use of muscle glycogen. Blood glucose levels and muscle glycogenolysis, glycolytic flux, PDH activation, and carbohydrate oxidation are all increased during exercise at higher, compared with moderate, exercise power outputs [32, 33].

### Decreased FFA Availability

The release of FFAs is decreased from adipose tissue at higher intensities of exercise, most likely due to reduced adipose tissue blood flow, resulting in decreased delivery of FFAs to the contracting muscle. When plasma FFA availability during heavy exercise (~80 % *V* O_2max_) was normalized to the levels observed during moderate-intensity exercise, FFA oxidation was increased, but not fully restored [10]. These data suggest that mechanisms within the muscle also limit fatty acid oxidation at higher exercise intensities.

### Downregulation of Fat Transport Across the Muscle Membrane?

Long chain fatty acid transport into skeletal muscle is a highly regulated process involving several LCFA transport proteins [18, 34–37]. Some of these transport proteins have also been shown to translocate to the plasma membrane during moderate exercise, acutely increasing the ability of skeletal muscle to transport FFAs into the cell [34, 36]. However, to date, the response of these transporters has not been examined during higher intensities of exercise, and it remains to be determined if FFA transport into muscle is compromised by a declining amount of LCFA transport proteins that are on the plasma membrane. While these direct measurements have not been made, one study reported that increasing the exercise intensity (from 40 to 80 % *V* O_2max_) reduced the uptake and oxidation of the transport-dependent LCFAs, but not the uptake and oxidation of membrane-independent medium chain LCFAs [26]. This suggested an inhibitory effect of increased glycolytic flux on the transport of FFAs at the plasma membrane. However, there could also be inhibition of FFA transport into mitochondria at the higher exercise intensity, as discussed in Sect. 6.6.

### Decreased IMTG Hydrolysis with Higher Intensities of Exercise?

The regulation of IMTG hydrolysis involves the key enzymes ATGL and HSL, which each release a FFA from the IMTG complex [22, 38]. Proteins known as perilipins also coat the lipid droplets and separate IMTG from ATGL and HSL, maintaining low rates of lipolysis. During moderate exercise, calcium- and epinephrine-related events phosphorylate HSL, and probably ATGL and AMP kinase phosphorylation of perilipin is involved in recruiting both HSL and ATGL to the lipid droplet, collectively enhancing rates of IMTG hydrolysis [3–41]. However, at higher power outputs it appears that AMP kinase phosphorylates additional sites on HSL that inhibit the phosphorylation by epinephrine and calcium, providing a potential mechanism for the lower rates of IMTG use [42]. It is not currently known if there are similar inhibitory events affecting ATGL and perilipin that may also decrease IMTG hydrolysis at higher exercise intensities and these remain to be investigated.

### Downregulation of Fat Transport Across the Mitochondrial Membranes?

The transport of LCFAs into mitochondria is a key step in regulating the overall rate that skeletal muscle can oxidize LCFAs. Historically, the regulation of fatty acid oxidation at the level of mitochondria has been solely attributed to the relationship between CPT I and malonyl-CoA (M-CoA) [43–45]. In rodent skeletal muscle at rest, M-CoA levels are highest and are believed to inhibit the transfer of LCFAs through the CPT complex into the mitochondria. When contractions occur, the M-CoA content decreased and relieved the inhibition on the CPT complex [44, 45]. However, while M-CoA has also been detected in human skeletal muscle, measurements at rest and during exercise have consistently demonstrated that the M-CoA content is unaffected by exercise at varying power outputs (35–100 % *V* O_2max_) and rates of fat oxidation [46–48]. Experiments with isolated mitochondria have also shown that mitochondrial fat oxidation can be increased without alterations in CPT I activity [49]. Finally, some exciting recent work using permeabilized skeletal muscle fibers has demonstrated that the ability of M-CoA to inhibit CPT I is dependent on the palmitoyl-CoA content [50]. This suggests that a robust increase in skeletal muscle palmitoyl-CoA content at the beginning of exercise could override any inhibitory effect of M-CoA and allow fatty acid transport and oxidation to proceed. These findings did not support a regulatory role for M-CoA content in fat oxidation during exercise in human skeletal muscle and suggested that the regulation of CPT I activity was more complex and that other transport proteins exist on mitochondrial membranes.

A plasma membrane fatty acid transport protein, fatty acid translocase (FAT)/CD36, was recently found on skeletal muscle mitochondrial membranes [51, 52]. FAT/CD36 appears to regulate mitochondrial fatty acid oxidation as fatty acid oxidation rates were lower in animals with no FAT/CD36 [19] and in human skeletal muscle, acute moderate-intensity exercise increases mitochondrial fatty acid oxidation and FAT/CD36 protein content [53], FAT/CD36 co-immunoprecipitates with CPT I [54], and exercise training increases the FAT/CD36 content on the mitochondria membranes to a greater extent than the increase in mitochondrial volume [55]. Evidence is mounting to suggest a regulatory role for FAT/CD36 in mitochondrial fatty acid oxidation, and while this research is novel there remains controversy as to the exact role of these proteins. However, Smith et al. [21] have proposed a theory describing the dual mechanism of action for skeletal muscle FAT/CD36 during exercise, acting at both the muscle and mitochondrial membranes to increase fatty acid transport into the muscle and mitochondria. Details suggest that FAT/CD36 is located on the outer mitochondrial membrane upstream of the acyl-CoA synthase enzyme. This location, in some unexplained manner, appears to facilitate the delivery of LCFA to this enzyme so it can proceed through the reaction and the CPT complex and into the mitochondria [56]. This research does not downplay the importance of the CPT complex in mitochondrial fatty acid transport, but rather indicates a complexity in the regulation of mitochondrial fatty acid transport not previously understood.

Another possibility for the downregulation of fat transport at the mitochondrial membranes relates to the small reductions in muscle pH that are associated with exercise at intense aerobic power outputs [33]. Studies in mitochondria isolated from resting human skeletal muscle showed that small decreases in pH from 7 to 6.8 caused large reductions in CPT I activity [51, 57]. This effect may also contribute to the decreased fatty acid transport and oxidation during exercise at higher power outputs. Surprisingly, attempts to demonstrate that increasing concentrations of calcium, and free ADP, AMP and inorganic phosphate, which occur at higher exercise intensities with increased carbohydrate use, also inhibited CPT I activity in mitochondria isolated from human skeletal muscle have not been successful [51].

### Does the Muscle Carnitine Content Limit Fatty Acid Transport into the Mitochondria?

It has also been proposed that the level of free carnitine may regulate mitochondrial FFA uptake during heavy dynamic exercise as it is a substrate for the CPT I reaction [31, 58, 59]. The carnitine content decreases as a function of increasing dynamic exercise intensity and increased glycolytic flux [31, 33]. The decreasing free carnitine level may limit the ability to transport FFAs into the mitochondria and ultimately limit FFA oxidation during dynamic exercise when glycolytic flux is high. However, it has been difficult to increase significantly the muscle carnitine content through means that an athlete could employ, and it is not currently known how much free carnitine is needed in the cytoplasm to maintain CPT I-mediated FFA transport into the mitochondria. It is interesting that the highest rates of FFA oxidation occur when the carnitine levels are already substantially lower than resting levels, but it is not known whether there is a lower limit that is reached once the exercise intensity is high. Carnitine is also not consumed in the transport process, but recycled back into the cytoplasm. While these factors make it unlikely that free carnitine availability limits FFA oxidation, no experimental model has been able to test whether this is a causal relationship in human skeletal muscle. However, a recent study reported a 21 % increase in muscle carnitine content following 6 months of oral carnitine supplementation. Surprisingly, the authors did not measure fat and carbohydrate oxidation rates and calculate the respiratory exchange ratio during a variety of aerobic power outputs as would be expected [60]. However, they did report glycogen sparing during moderate exercise following carnitine supplementation, which is consistent with increased fat oxidation.

To summarize, it appears that the regulation of fat metabolism in skeletal muscle has been designed to reduce the reliance on fat-derived ATP at intense aerobic power outputs. This may be due to the greater ATP produced/oxygen consumed (P/O ratio) when carbohydrate is the substrate. As high power outputs are reached and the ability to deliver oxygen from the environment to the muscle mitochondria nears maximum capacity, it makes teleological sense to rely on carbohydrate as a fuel. Several sites of downregulation may exist during high-intensity aerobic exercise, including decreased blood flow to adipose tissue, decreased release of FFAs into the plasma and decreased delivery of FFAs to skeletal muscle, decreased FFA transport into muscle, decreased breakdown of IMTGs and decreased delivery of FFAs to skeletal muscle mitochondria, and decreased FFA transport into mitochondria.

## Conclusions

Fat and carbohydrate are important fuels for aerobic exercise. However, at a given exercise intensity and metabolic demand, there can be reciprocal shifts in the proportions of carbohydrate and fat that are oxidized. The interaction between carbohydrate and fatty acid oxidation is dependent on the intracellular and extracellular metabolic environments. The availability of substrate (both from inside and outside of the muscle), and the exercise intensity and duration can affect these environments. Experiments 50 years ago proposed intracellular mechanisms that could explain the ability of increasing fat availability to downregulate carbohydrate metabolism in the heart and diaphragm muscle. More recent work extended these findings to peripheral skeletal muscle. However, the regulation of fat metabolism in human skeletal muscle during exercise in the face of increasing carbohydrate availability and exercise intensity was not well studied. The past 10 years has seen incredible progress in the understanding of fat metabolism in skeletal muscle, and it is now realized that the regulation is complex and involves many sites of control. These include the transport of FFAs into the muscle cell, the binding and transport of FFAs in the cytoplasm, the regulation of IMTG synthesis and breakdown, and the transport of fat into the mitochondria. The discovery of proteins that assist in transporting fat across the plasma and mitochondrial membranes, the ability of these proteins to translocate to the membranes during exercise, and the newly discovered roles of ATGL and HSL in regulating and skeletal muscle lipolysis are examples of recent discoveries. This information has enabled a more complete understanding of the relationship between fat and carbohydrate metabolism during exercise and mechanistic proposals to explain the downregulation of fat metabolism that occurs when carbohydrate availability is increased and when moving from moderate to intense aerobic exercise.
